# Association between state-level socioeconomic vulnerability and access to community water fluoridation in the United States

**DOI:** 10.1371/journal.pone.0360293

**Published:** 2026-11-11

**Authors:** Donald L. Chi, JoAnna M. Scott, Deeksha Nemawarkar

**Affiliations:** 1 Department of Oral Health Sciences, University of Washington School of Dentistry, Seattle, Washington, United States of America; 2 Department of Health Systems and Population Health, University of Washington School of Public Health, Seattle, Washington, United States of America; 3 School of Medicine and Dentistry, Griffith University, Gold Coast, Australia,; 4 Research and Graduate Programs, University of Missouri Kansas City, School of Dentistry, Kansas City, Missouri, United States of America; Indiana University School of Dentistry, UNITED STATES OF AMERICA

## Abstract

To evaluate associations between state-level socioeconomic vulnerability and community water fluoridation (CWF) in the U.S. Data from calendar year 2022 were drawn from the U.S. Census Bureau to develop four state-level markers of socioeconomic vulnerability (percentages non-white, Hispanic/Latino, below federal poverty level, and <high school education) and the U.S. Centers for Disease Control and Prevention’s Water Fluoridation Reporting System to assess mean state-level CWF percent. Fractional logit regression models with robust standard errors, weighted by state population, were used to assess non-adjusted associations between each socioeconomic vulnerability marker and CWF (α < 0.05). Poverty-adjusted models were also run. The mean state-level CWF was 72.8% (SD = 20.0%; range:8.5%−100%). In the non-adjusted models, only state-level percentages of non-white and Hispanic/Latino individuals were significantly associated with lower state-level CWF percent. In the poverty-adjusted models, higher percentages of non-whites, Hispanic/Latinos, and individuals who did not complete high school at the state-level were associated with a significantly lower state-level CWF percent (p = 0.01, p < 0.01, and p < 0.01, respectively). Individuals living in more socioeconomically vulnerable states in the U.S. may face inequities in CWF access.

## Introduction

Community water fluoridation (CWF) is a safe and cost-effective public health strategy that prevents tooth decay (also called cavities or dental caries) [1,2]. CWF prevents tooth decay by remineralizing tooth enamel and inhibiting enamel demineralization. Dental caries is a multifactorial disease caused by oral microbiome dysbiosis, host susceptibility, and behavioral factors, of which added sugars in the diet and insufficient topical fluoride exposure are the leading contributors [3]. Numerous studies have highlighted the importance of CWF, particularly in socioeconomically vulnerable communities at increased risk for tooth decay, which helps dampen the effects of dietary sugars on oral health [4–8].

CWF availability may be associated with factors like income, race, and ethnicity. A World Health Organization (WHO) study reported that higher-income countries were more likely to have country-wide CWF initiatives than lower-income countries, which is consistent with findings from Australia and Brazil that larger numbers of higher-income individuals lived in areas with CWF [9–11]. Conversely, non-white U.S. children were 39% to 70% more likely to live in a high CWF county than white children which is consistent with findings from a more recent U.S. county-level analysis from Washington state indicating that greater racial and ethnic diversity was associated with significantly greater county-level CWF prevalence [12,13]. However, this latter study also reported that counties in Washington state with larger proportions of individuals in poverty had significantly lower CWF prevalence [13]. A Canadian study showed that rural areas have significantly lower CWF rates than urban areas [14]. This finding aligns with a 2011 U.S. Rural Health Research Center report, which found that individuals living in rural communities are significantly less likely to have access to CWF than those in urban areas, in part because individuals in rural populations rely on private wells or smaller water systems for which fluoridation may not be possible because of significantly higher per-person costs for fluoridation [15].

While it is widely accepted in public health that CWF helps promote oral health equity by reducing barriers to public sources of fluoride made available through public water systems, there are currently limited U.S. studies on whether socioeconomically vulnerable communities have equitable access to CWF [7,13]. This is particularly worrisome given the disproportionately negative health consequences borne by low-income and minoritized communities that do not have access to CWF or may be at risk of losing access to CWF [16]. Moreover, statements that question the safety and importance of CWF made by Robert F. Kennedy, Jr., the current Secretary of the U.S. Department of Health and Human Services, have jeopardized CWF as a public health measure [17]. His remarks have resulted in immediate policy actions: both Utah and Florida enacted statewide bans on CWF within the first 100 days of the new administration, while at least 21 other states have introduced bills to eliminate CWF, further putting at risk an important source of community-level prevention [18,19].

The goal of this study was to evaluate the associations between state-level socioeconomic vulnerability markers – race, ethnicity, poverty, and education – and CWF in the U.S [13]. Based on the premise that socioeconomic vulnerability is associated with an increased risk for tooth decay and need for fluoride [6], and prior national and county-level data indicating greater CWF prevalence in areas with higher proportions of non-white populations [12,13], we hypothesize positive associations between socioeconomic vulnerability markers (i.e., non-white race, Hispanic/Latino ethnicity, having an income below the federal poverty level, and not graduated from high school) and CWF at the state-level.

## Materials and Methods

This was an ecological cross-sectional secondary analysis of publicly available data. The outcome was mean state-level CWF percent in 2022, obtained from the U.S. Centers for Disease Control and Prevention’s Water Fluoridation Reporting System (CDC WFRS) [20]. The predictor variables were four vulnerability markers adopted from a previous study and derived from 2022 state-level data from the U.S. Census Bureau: % of population non-white, % Hispanic/Latino, % below the federal poverty level, and % age 25 and older not graduated from high school [6,13,21]. Per U.S. Census summary tables, individuals identifying as Hispanic/Latino were included in the % non-white population. All variables were percentages and treated as continuous variables in the models. Figures were created in R (version 4.6.0) [22]; using the ggplot2 [23], usmap [24], and sf packages [25].

Fractional response regression (logit model) with robust standard errors was used to evaluate the non-adjusted associations among the four vulnerability markers and the mean state-level % of the population with CWF [26,27]. Fractional logit regression is appropriate for percentage outcomes (values range between 0 and 1 and may include 0 or 1), and does not require distributional assumptions beyond the correct specification of the conditional mean [26,27]. Because coefficients from fractional logit models are on the log-odds scale and not easily interpretable, average marginal effects (AMEs) were reported as the primary effect measure. AMEs represent the average change in the predicted percent of state-level CWF associated with a one percent change in the predictor. Models were weighted by the state population. As a sensitivity analysis, a second set of models were run with each of the three vulnerability markers and state-level poverty level included as a confounder. Adequate specification of the conditional mean model was found for all regression models. All analyses were conducted using Stata 18.0 (StataCorp LLC, College Station, TX 77845 USA) with the significance level set to 0.05. Because the study was based on publicly available data, there was no institutional review board approval required.

## Results

The mean state-level CWF was 72.8% (SD = 20.0%; range: 8.5% to 100%) (Fig 1). The mean (SD) percentage of non-whites was 39.1 (13.8) (Fig 2), and of Hispanic/Latinos was 19.1 (13.4) (Fig 3). The mean percentage of individuals below the federal poverty level was 12.6 (2.0) (Fig 4). Among those aged 25 and over, the mean percentage with less than a high school education was 10.4% (2.7) (Fig 5).

**Fig 1:**
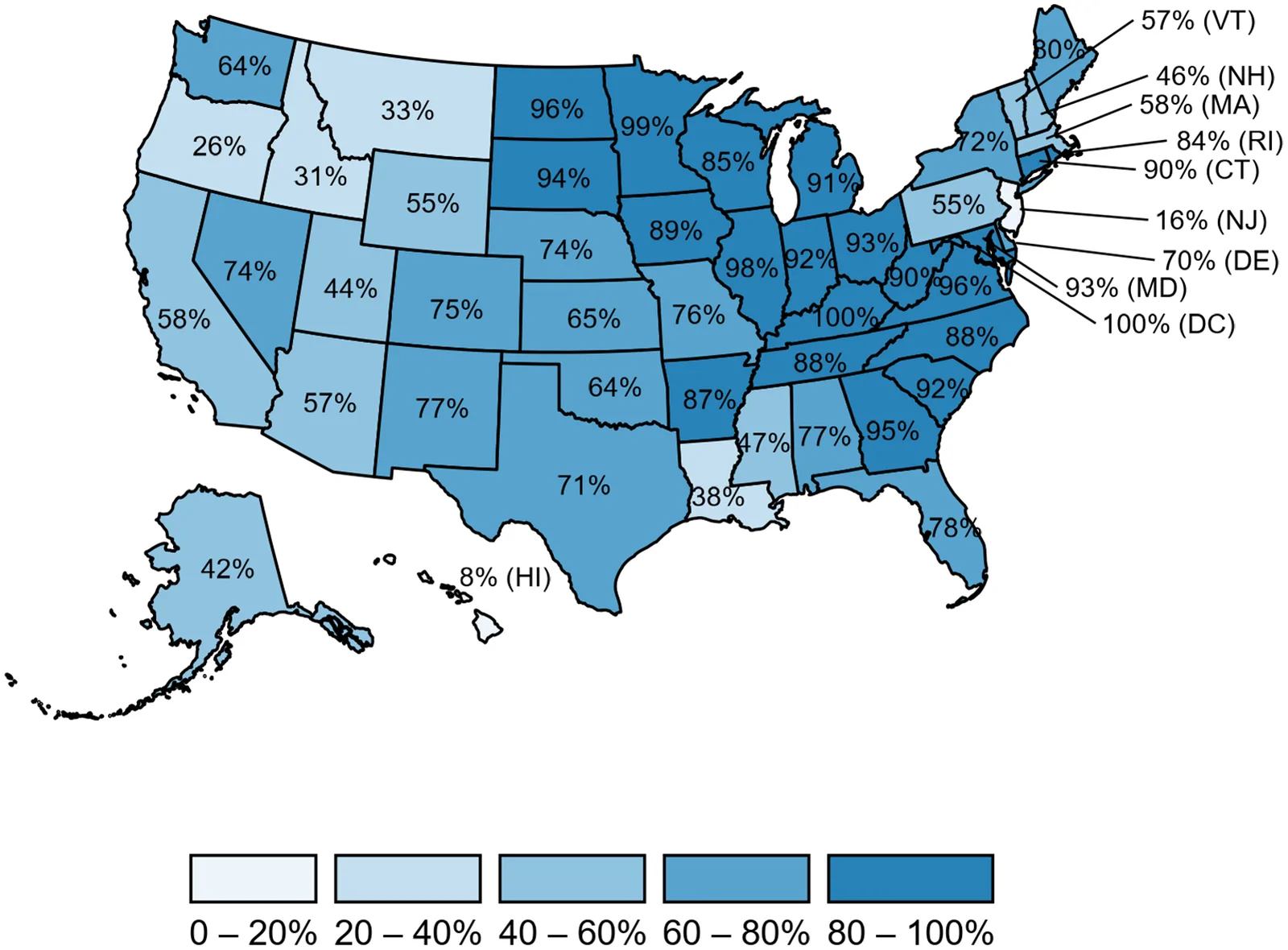
State-level percentage of the population served by community water systems receiving fluoridated water, United States, 2022. Base map: U.S. Census Bureau public-domain Cartographic Boundary Files (1:20,000,000; https://urldefense.com/v3/__https://www.census.gov/geographies/mapping-files/time-series/geo/cartographic-boundary.html__;!!K-Hz7m0Vt54!nSZa_hs2s6PEzIukCZPjnivEj9R9Wq4n3QRmKpvW2DQdPmfNA5XmH3UYBynw__as-MusbE6szReb$), accessed via the R package usmap.

**Fig 2:**
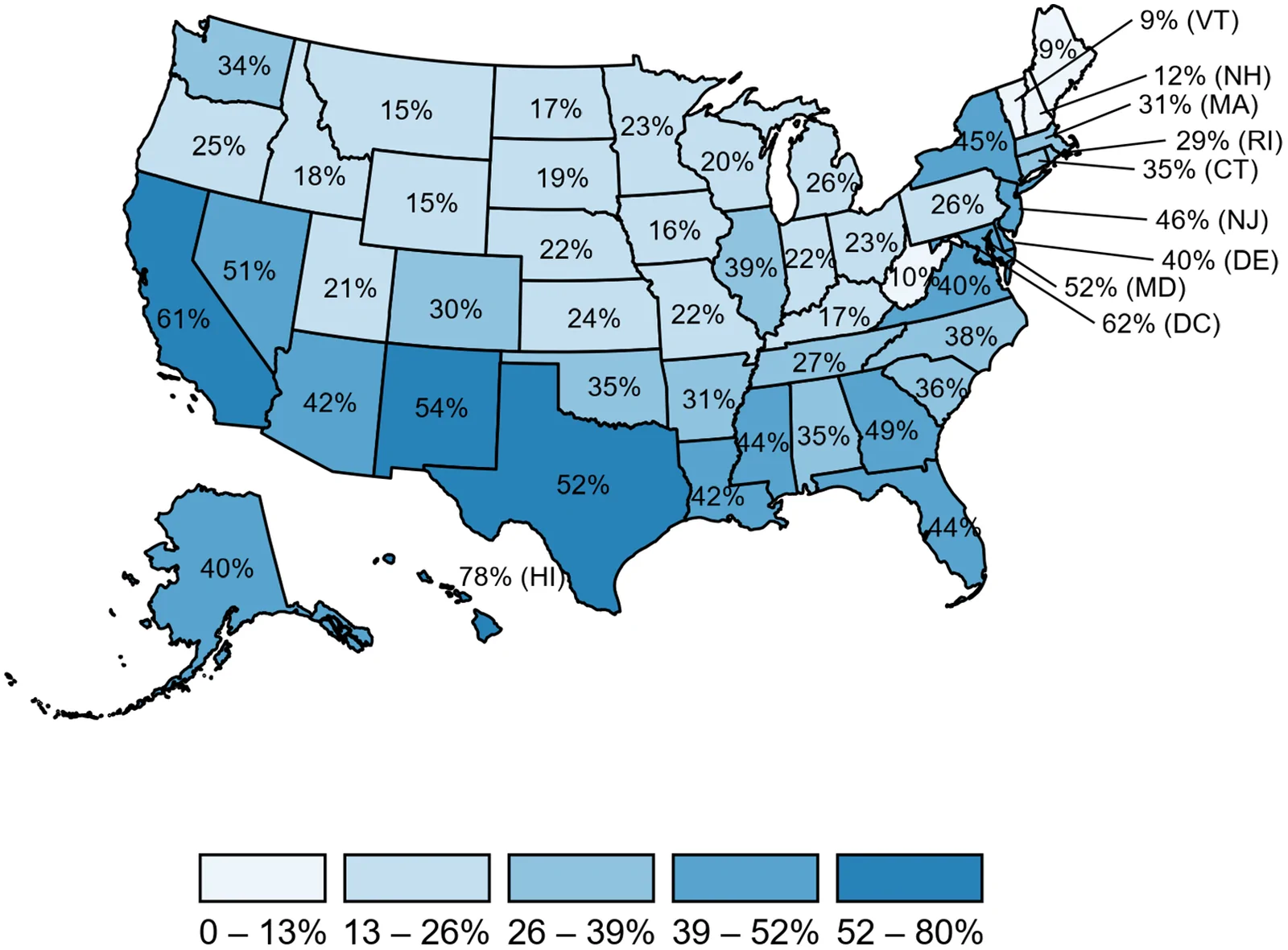
State-level percentage of non-white individuals, United States, 2022. Base map: U.S. Census Bureau public-domain Cartographic Boundary Files (1:20,000,000; https://urldefense.com/v3/__https://www.census.gov/geographies/mapping-files/time-series/geo/cartographic-boundary.html__;!!K-Hz7m0Vt54!nSZa_hs2s6PEzIukCZPjnivEj9R9Wq4n3QRmKpvW2DQdPmfNA5XmH3UYBynw__as-MusbE6szReb$), accessed via the R package usmap.

**Fig 3:**
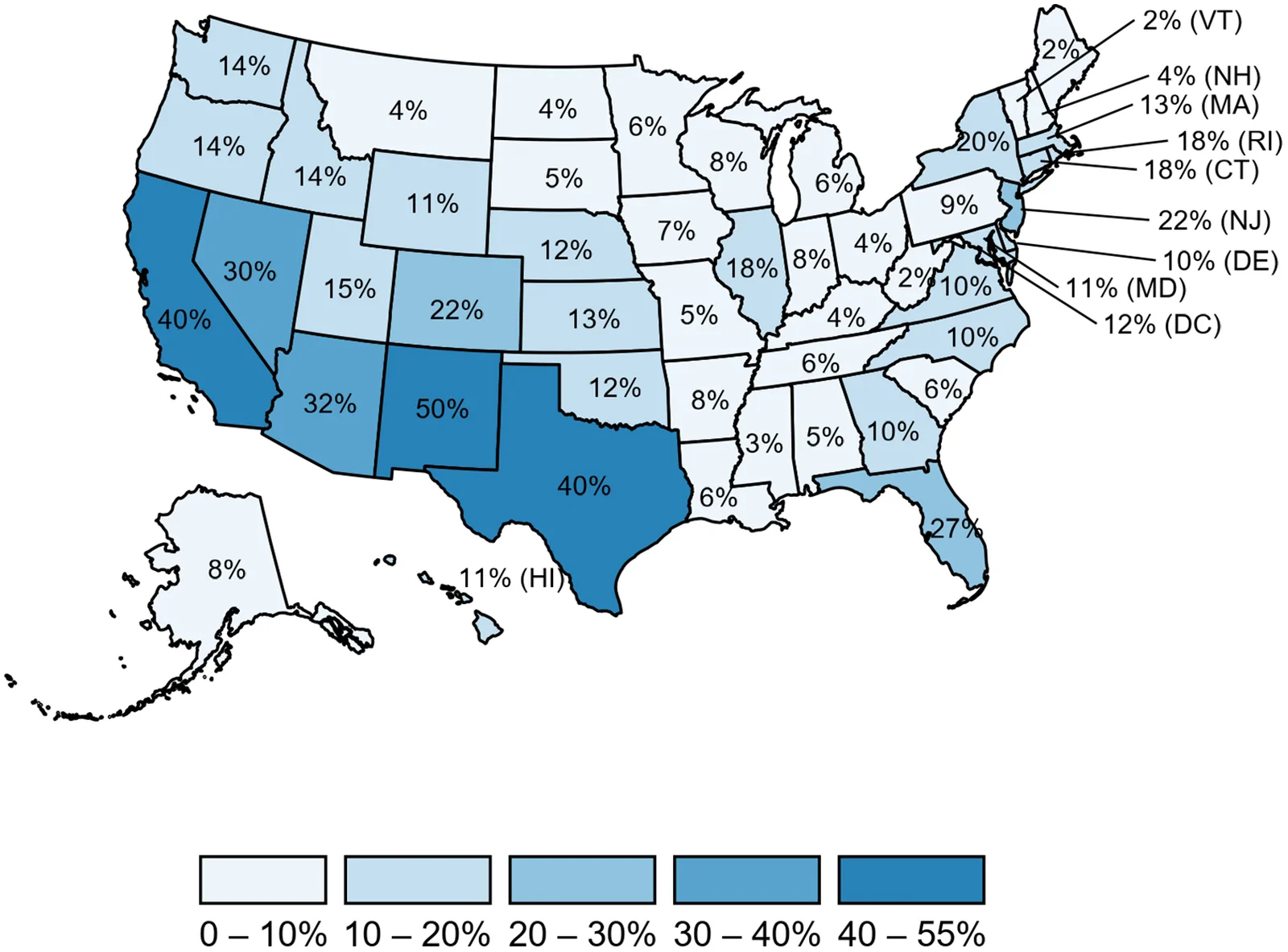
State-level percentage of Hispanic/Latino individuals, United States, 2022. Base map: U.S. Census Bureau public-domain Cartographic Boundary Files (1:20,000,000; https://urldefense.com/v3/__https://www.census.gov/geographies/mapping-files/time-series/geo/cartographic-boundary.html__;!!K-Hz7m0Vt54!nSZa_hs2s6PEzIukCZPjnivEj9R9Wq4n3QRmKpvW2DQdPmfNA5XmH3UYBynw__as-MusbE6szReb$), accessed via the R package usmap.

**Fig 4:**
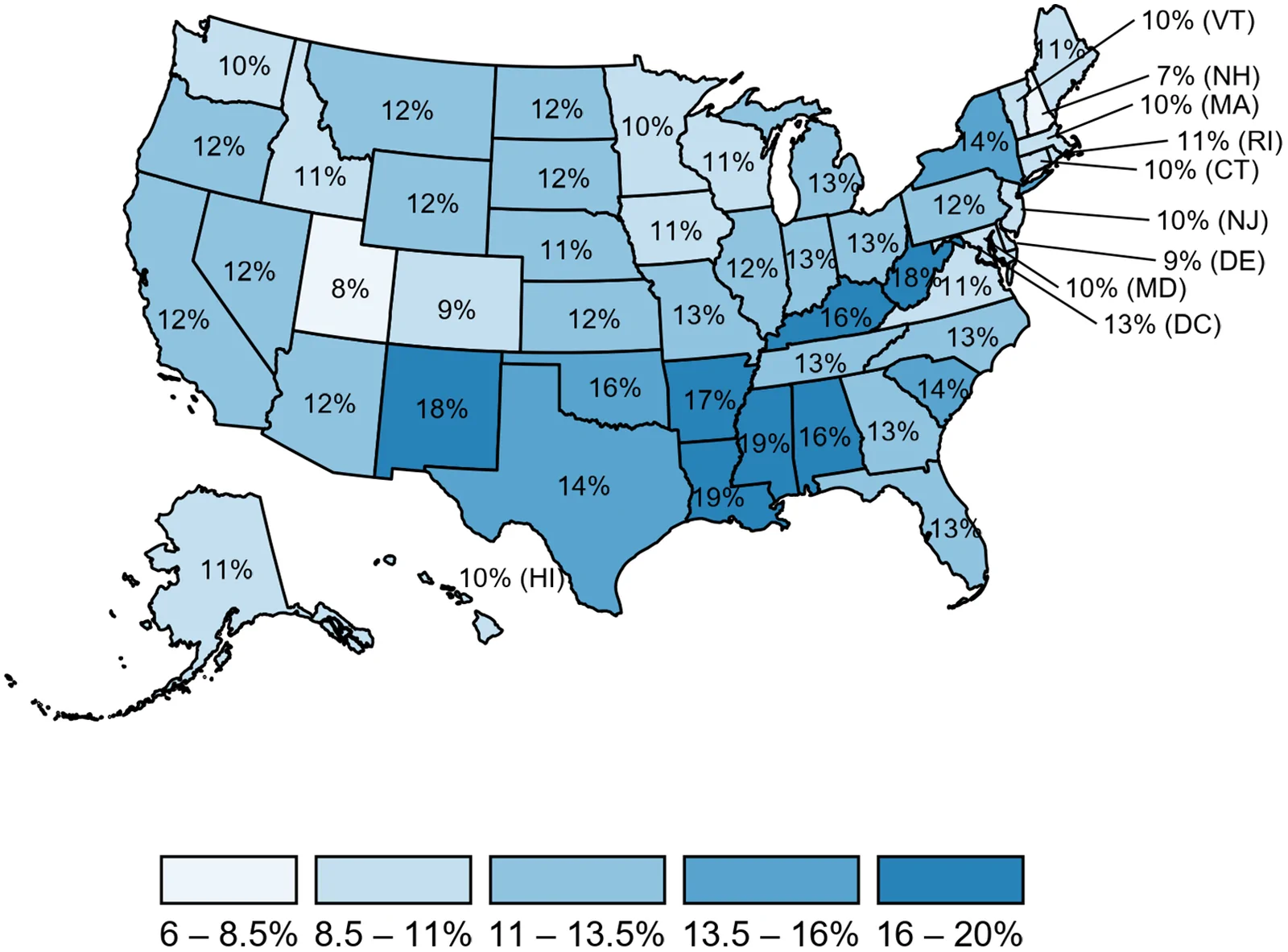
State-level percentage of individuals below the federal poverty level, United States, 2022. Base map: U.S. Census Bureau public-domain Cartographic Boundary Files (1:20,000,000; https://urldefense.com/v3/__https://www.census.gov/geographies/mapping-files/time-series/geo/cartographic-boundary.html__;!!K-Hz7m0Vt54!nSZa_hs2s6PEzIukCZPjnivEj9R9Wq4n3QRmKpvW2DQdPmfNA5XmH3UYBynw__as-MusbE6szReb$), accessed via the R package usmap.

**Fig 5:**
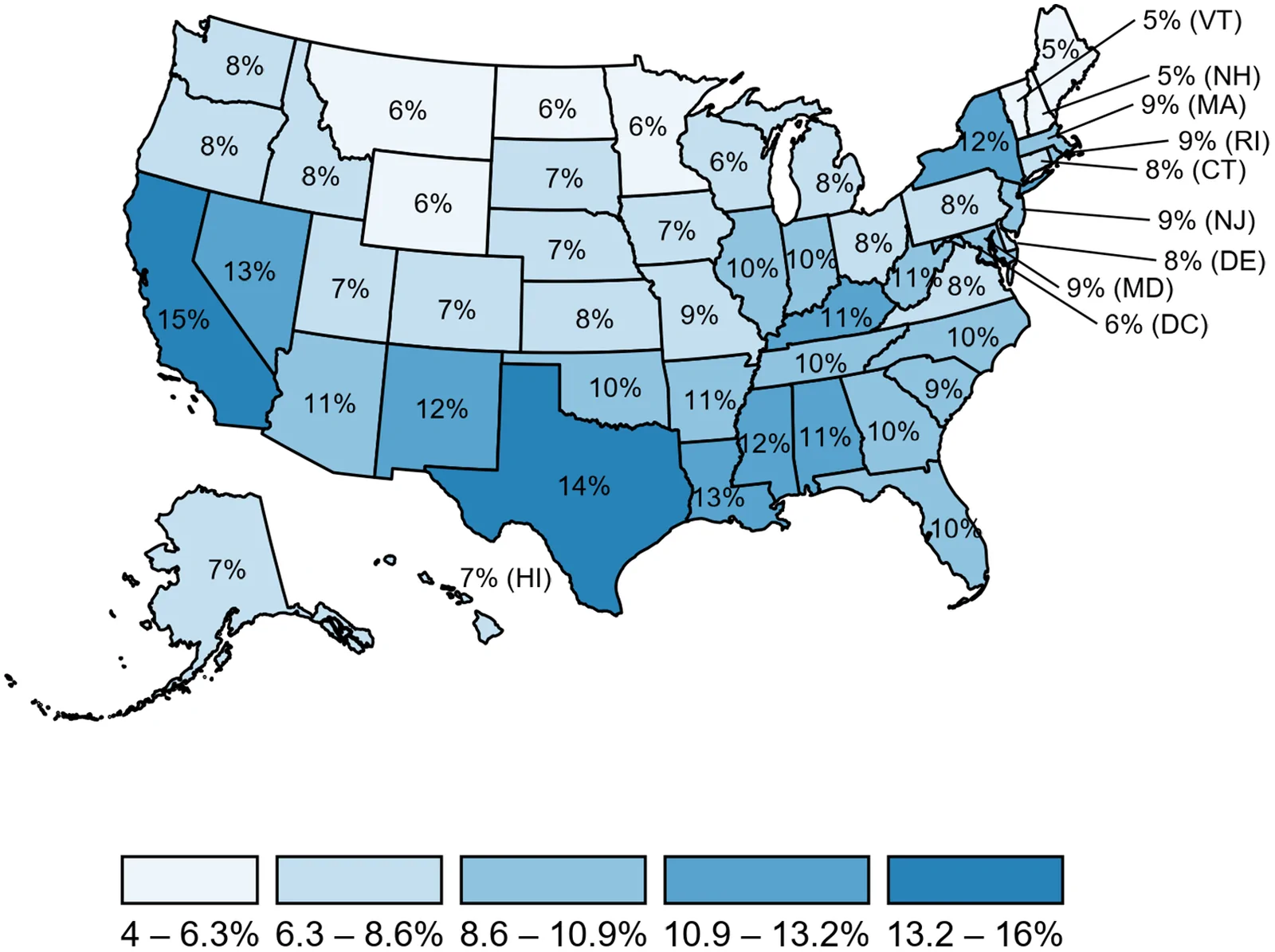
State-level percentage of individuals age 25 years and older who did not complete high school, United States, 2022. Base map: U.S. Census Bureau public-domain Cartographic Boundary Files (1:20,000,000; https://urldefense.com/v3/__https://www.census.gov/geographies/mapping-files/time-series/geo/cartographic-boundary.html__;!!K-Hz7m0Vt54!nSZa_hs2s6PEzIukCZPjnivEj9R9Wq4n3QRmKpvW2DQdPmfNA5XmH3UYBynw__as-MusbE6szReb$), accessed via the R package usmap.

State-level percentages of non-white and Hispanic/Latino individuals were significantly associated with CWF in the adjusted models whereby higher state-level percentages of non-white or Hispanic/Latinos were associated with significantly lower mean state-level % of the population with CWF (Table 1). Specifically, a 10% increase in the state-level percentage of non-white or Hispanic/Latino was associated on average with a 3.9% and 4.6% reduction in state-level CWF, respectively (p = 0.02 and p < 0.01). In the adjusted models, state-level poverty and education were not associated with state-level CWF (p = 0.52 and p = 0.051). After adjusting for poverty, all three state-level vulnerability markers were significantly associated with state-level CWF whereby race and ethnicity had similar results as in the adjusted models and education level became significantly associated with CWF. A 10% increase in the percentage of 25-year-olds or older with a less than high school education, on average was associated with a 23.9% decrease in state-level CWF (Table 1).

**Table 1:** Associations between four state-level vulnerability markers and Community Water Fluoridation percent in the U.S. based on fractional logit regression models with robust standard errors and weighted by state population.

State-level vulnerability marker	Non-adjusted fractional logit regression models			Poverty-adjusted fractional logit models		
	Average Marginal Effects*	95% CI	p-value	Average Marginal Effects*	95% CI	p-value
**% non-white**	−0.39	(−0.73, −0.06)	0.02	−0.41	(−0.74, −0.09)	0.01
**% Hispanic/Latino**	−0.46	(−0.76, −0.17)	<0.01	−0.48	(−0.76, −0.19)	<0.01
**% below federal poverty level**	1.12	(−2.29, 4.53)	0.52	--	--	--
**% age 25 years and older who did not complete high school**	−1.61	(−3.22, 0.01)	0.051	−2.39	(−3.46, −1.32)	<0.01

* Average Marginal Effects (AMEs) represent the average change in the predicted proportion of Community Water Fluoridation (CWF) associated with a one percent change in each vulnerability marker

## Discussion

In this study, we examined the associations between state-level socioeconomic vulnerability markers (race, ethnicity, poverty, and education) and CWF in the U.S. In the models that adjusted for state-level poverty, all three remaining vulnerability markers were significantly associated with state-level CWF, but in the opposite direction as originally hypothesized. States with higher percentages of non-whites, Hispanic/Latinos, or individuals who did not complete high school had a significantly lower percentages of their population with access to CWF, which is inconsistent with previous national data on U.S. children and recent county-level findings from Washington state [12,13]. These new findings raise concerns about the extent to which CWF is equitably available to both vulnerable and less vulnerable populations.

Because CWF is associated with reduced dental caries risk, less CWF in states with higher proportions of socioeconomically vulnerable individuals may contribute to a higher burden of dental disease in these populations [5,6]. This is consistent with national data documenting a relatively high prevalence of untreated tooth decay among U.S. Latino populations, though future studies directly examining the relationship between CWF access and dental caries outcomes in Hispanic/Latino communities are needed [28,29]. Similarly, past studies have reported higher dental caries burden in racial minorities and individuals with less educational attainment [28,30]. The current findings raise questions and concerns about the assumption that socioeconomically vulnerable and minoritized communities in the U.S. have equal or adequate access to CWF as others.

There is heterogeneity in the way in which CWF decisions are made in the U.S. At least 12 states have mandatory CWF laws for water systems serving communities above a certain population size, including California, Georgia, Illinois, and Ohio [19]. For instance, a 1995 California law requires water systems serving communities larger than 10,000 to fluoridate community water, consistent with U.S. CDC guidelines [31]. These 12 states have a wide range of CWF % (37.9% to 99.7%), which also suggests that mandatory laws alone cannot guarantee CWF. In the remaining states, fluoridation is determined at the community level – by elected city officials, water utility boards, private water companies, and sometimes through public referenda [32]. These differential legislative and governance structures suggest that local political and administrative factors may be important determinants of CWF, underscoring the importance of advocating for state-level as well as local CWF policies that protect the oral health of the public.

Little is known about the extent to which socioeconomically vulnerable communities are concerned about CWF. Previous studies have highlighted concerns that marginalized individuals have about tap water in general, which may supersede any support or concern about fluoride [33,34]. Beyond perceptions of tap water safety, access to CWF does not guarantee fluoride exposure at the individual level, as increasing use of bottled water and point-of-use reverse osmosis filtration, which removes fluoride from drinking water, may limit the benefits of CWF even among those in fluoridated communities [33,34]. Additionally, approximately 43 million Americans, or 15% of the U.S. population, rely on private wells that are not subject to CWF, further limiting population-level fluoride exposure [35]. Future studies should continue to clarify the need to address latent concerns about tap water safety first and then if tap water is acceptable how community-level preventive efforts like CWF can be marketed as a public health benefit to increase its acceptability. The findings underscore the need for additional research on ways to improve access to CWF, especially for individuals living in vulnerable and minoritized communities. These scientific efforts are part of a comprehensive public health strategy aimed at preventing tooth decay and reducing oral health inequities that disproportionately affect underserved populations.

The study had three main limitations. First, the analyses were cross-sectional, which precludes any assumptions of causation between vulnerability markers and CWF. Second, the analysis was conducted at the state-level. We did not include a state-level rural vs. urban variable in our models, even though studies have shown that CWF is significantly lower in rural areas, which future studies should explore further [14,15]. Associations observed at the state-level may not necessarily apply to individuals. Unknown is the extent to which these findings can be generalized to smaller geographic groupings of marginalized and socioeconomically vulnerable individuals (e.g., counties). Third, the reasons for our findings are unknown. Future qualitative studies are needed to examine factors, such as geographic distribution, income, and rurality, that may impact CWF access in socioeconomically vulnerable communities. These efforts are necessary to fully interpret the reasons why states with higher proportions of non-whites, Hispanic/Latinos, and individuals with lower educational attainment had lower CWF prevalence.

## Conclusions

Individuals living in more socioeconomically vulnerable states in the U.S. may face inequitable access to CWF. These findings underscore the importance of public health strategies aimed at ensuring equitable access to CWF especially for those in socioeconomically communities.
