# Supplementary material for: Association between state-level socioeconomic vulnerability and access to community water fluoridation in the United States
Source: PLoS One. 2026 Nov 11;21(11):e0360293. doi: 10.1371/journal.pone.0360293 (PMC13754221; doi:10.1371/journal.pone.0360293)
Supplement: Supplementary file 1 [file pone.0360293.s001.docx]

Plos One Article: Association between state-level socioeconomic vulnerability and access to community water fluoridation in the United States.

Journal Requirements:

When submitting your revision, we need you to address these additional requirements.

1.Please ensure that your manuscript meets PLOS ONE's style requirements, including those for file naming. The PLOS ONE style templates can be found at

[https://journals.plos.org/plosone/s/file?id=wjVg/PLOSOne_formatting_sample_main_body.pdf](https://urldefense.com/v3/__https:/journals.plos.org/plosone/s/file?id=wjVg*PLOSOne_formatting_sample_main_body.pdf__;Lw!!K-Hz7m0Vt54!lbLw9oBzBxIT_8rfcfT_YaykAWLEQLO4OP5ji1G3gDpwPRf55JySUgCU6DWaK7IyUKyj3nwWWTPT$) and

[https://journals.plos.org/plosone/s/file?id=ba62/PLOSOne_formatting_sample_title_authors_affiliations.pdf](https://urldefense.com/v3/__https:/track.editorialmanager.com/CL0/https:*2F*2Fjournals.plos.org*2Fplosone*2Fs*2Ffile*3Fid=ba62*2FPLOSOne_formatting_sample_title_authors_affiliations.pdf/1/010f019e91e09c44-60246e19-f83a-4cd8-83fd-760df2eaa5d6-000000/pbdfxZKhiM1vWN6emkHhhgW5JOW87-UD_jnDcIMIdZE=258__;JSUlJSUlJQ!!K-Hz7m0Vt54!lbLw9oBzBxIT_8rfcfT_YaykAWLEQLO4OP5ji1G3gDpwPRf55JySUgCU6DWaK7IyUKyj3iWJ8b6W$)

**Done.**

2. In the online submission form you indicate that your data is not available for proprietary reasons and have provided a contact point for accessing this data. Please note that your current contact point is a co-author on this manuscript. According to our Data Policy, the contact point must not be an author on the manuscript and must be an institutional contact, ideally not an individual. Please revise your data statement to a non-author institutional point of contact, such as a data access or ethics committee, and send this to us via return email. Please also include contact information for the third party organization, and please include the full citation of where the data can be found.

**We apologize for the confusion. The data are publicly available. They have been provided.**

3. Please include a caption for Figure 1.

**A caption for Figure 1 has been provided.**

4. We note that Figure 1 in your submission contain map images which may be copyrighted. All PLOS content is published under the Creative Commons Attribution License (CC BY 4.0), which means that the manuscript, images, and Supporting Information files will be freely available online, and any third party is permitted to access, download, copy, distribute, and use these materials in any way, even commercially, with proper attribution. For these reasons, we cannot publish previously copyrighted maps or satellite images created using proprietary data, such as Google software (Google Maps, Street View, and Earth). For more information, see our copyright guidelines: [http://journals.plos.org/plosone/s/licenses-and-copyright](https://urldefense.com/v3/__http:/journals.plos.org/plosone/s/licenses-and-copyright__;!!K-Hz7m0Vt54!lbLw9oBzBxIT_8rfcfT_YaykAWLEQLO4OP5ji1G3gDpwPRf55JySUgCU6DWaK7IyUKyj3p9VdFxb$).

We require you to either (1) present written permission from the copyright holder to publish these figures specifically under the CC BY 4.0 license, or (2) remove the figures from your submission:

 1. You may seek permission from the original copyright holder of Figure 1 to publish the content specifically under the CC BY 4.0 license.

 We recommend that you contact the original copyright holder with the Content Permission Form ([http://journals.plos.org/plosone/s/file?id=7c09/content-permission-form.pdf](https://urldefense.com/v3/__http:/journals.plos.org/plosone/s/file?id=7c09*content-permission-form.pdf__;Lw!!K-Hz7m0Vt54!lbLw9oBzBxIT_8rfcfT_YaykAWLEQLO4OP5ji1G3gDpwPRf55JySUgCU6DWaK7IyUKyj3skyMmep$)) and the following text:

“I request permission for the open-access journal PLOS ONE to publish XXX under the Creative Commons Attribution License (CCAL) CC BY 4.0 ([http://creativecommons.org/licenses/by/4.0/](https://urldefense.com/v3/__http:/creativecommons.org/licenses/by/4.0/__;!!K-Hz7m0Vt54!lbLw9oBzBxIT_8rfcfT_YaykAWLEQLO4OP5ji1G3gDpwPRf55JySUgCU6DWaK7IyUKyj3m-ibxS_$)). Please be aware that this license allows unrestricted use and distribution, even commercially, by third parties. Please reply and provide explicit written permission to publish XXX under a CC BY license and complete the attached form.”

Please upload the completed Content Permission Form or other proof of granted permissions as an 'Other' file with your submission.

 In the figure caption of the copyrighted figure, please include the following text: “Reprinted from [ref] under a CC BY license, with permission from [name of publisher], original copyright [original copyright year].”

**We have replaced Figure 1 with a new Figure 1 that is not copyrighted.**

 2. If you are unable to obtain permission from the original copyright holder to publish these figures under the CC BY 4.0 license or if the copyright holder’s requirements are incompatible with the CC BY 4.0 license, please either i) remove the figure or ii) supply a replacement figure that complies with the CC BY 4.0 license. Please check copyright information on all replacement figures and update the figure caption with source information. If applicable, please specify in the figure caption text when a figure is similar but not identical to the original image and is therefore for illustrative purposes only.

The following resources for replacing copyrighted map figures may be helpful:

 USGS National Map Viewer (public domain): [http://viewer.nationalmap.gov/viewer/](https://urldefense.com/v3/__http:/viewer.nationalmap.gov/viewer/__;!!K-Hz7m0Vt54!lbLw9oBzBxIT_8rfcfT_YaykAWLEQLO4OP5ji1G3gDpwPRf55JySUgCU6DWaK7IyUKyj3psUrpaF$)

The Gateway to Astronaut Photography of Earth (public domain): [http://eol.jsc.nasa.gov/sseop/clickmap/](https://urldefense.com/v3/__http:/eol.jsc.nasa.gov/sseop/clickmap/__;!!K-Hz7m0Vt54!lbLw9oBzBxIT_8rfcfT_YaykAWLEQLO4OP5ji1G3gDpwPRf55JySUgCU6DWaK7IyUKyj3vzKfd1A$)

Maps at the CIA (public domain): [https://www.cia.gov/library/publications/the-world-factbook/index.html](https://urldefense.com/v3/__https:/www.cia.gov/library/publications/the-world-factbook/index.html__;!!K-Hz7m0Vt54!lbLw9oBzBxIT_8rfcfT_YaykAWLEQLO4OP5ji1G3gDpwPRf55JySUgCU6DWaK7IyUKyj3oIVYHWB$) and [https://www.cia.gov/library/publications/cia-maps-publications/index.html](https://urldefense.com/v3/__https:/www.cia.gov/library/publications/cia-maps-publications/index.html__;!!K-Hz7m0Vt54!lbLw9oBzBxIT_8rfcfT_YaykAWLEQLO4OP5ji1G3gDpwPRf55JySUgCU6DWaK7IyUKyj3hD7Sm16$)

NASA Earth Observatory (public domain): [http://earthobservatory.nasa.gov/](https://urldefense.com/v3/__http:/earthobservatory.nasa.gov/__;!!K-Hz7m0Vt54!lbLw9oBzBxIT_8rfcfT_YaykAWLEQLO4OP5ji1G3gDpwPRf55JySUgCU6DWaK7IyUKyj3jWPqsB7$)

Landsat: [http://landsat.visibleearth.nasa.gov/](https://urldefense.com/v3/__http:/landsat.visibleearth.nasa.gov/__;!!K-Hz7m0Vt54!lbLw9oBzBxIT_8rfcfT_YaykAWLEQLO4OP5ji1G3gDpwPRf55JySUgCU6DWaK7IyUKyj3rvpZ0Wa$)

USGS EROS (Earth Resources Observatory and Science (EROS) Center) (public domain): [http://eros.usgs.gov/#](https://urldefense.com/v3/__http:/eros.usgs.gov/*__;Iw!!K-Hz7m0Vt54!lbLw9oBzBxIT_8rfcfT_YaykAWLEQLO4OP5ji1G3gDpwPRf55JySUgCU6DWaK7IyUKyj3i09IXn3$)

Natural Earth (public domain): [http://www.naturalearthdata.com/](https://urldefense.com/v3/__http:/www.naturalearthdata.com/__;!!K-Hz7m0Vt54!lbLw9oBzBxIT_8rfcfT_YaykAWLEQLO4OP5ji1G3gDpwPRf55JySUgCU6DWaK7IyUKyj3igmkI-F$)

**A replacement Figure 1 has been provided in the revised manuscript submission.**

**Comments to the Author**

5. Review Comments to the Author

Reviewer #1: The authors of the study titled “Association between state-level socioeconomic vulnerability and community water fluoridation in the United States” conducted a secondary analysis that provides a valuable contribution to the literature exploring socioeconomic factors that may limit access to community water fluoridation, particularly in minority populations. Below I provide comments for the authors' consideration prior to publication of this work in the journal:

Introduction

Lines 58-60: The authors emphasize the importance of CWF in socioeconomically vulnerable communities at increased risk of dental caries, but with a main framing of the protective effects against dietary sugars. A more updated multifactorial focus of dental caries, expanding beyond dietary sugars, should be incorporated.

**Thank you for this suggestion. We provide additional text and added a new reference to provide context on the multifactorial etiology of dental caries.**

Lines 78-79: The authors state “there are currently no U.S. studies on whether socioeconomically vulnerable communities have equitable access to CWF”. However, the following studies have been previously reported:

Chi, D. L., Guinn, S., Shands, M. E., Nemawarkar, D., Hill, C. M., Mayhle, M., Do, T. T., Li, S., & Panchal, S. (2024). Socioeconomic vulnerability and access to community water fluoridation in Washington. Journal of the American Dental Association (1939), 155(9), 747–754.e2. [https://doi.org/10.1016/j.adaj.2024.05.011](https://urldefense.com/v3/__https:/doi.org/10.1016/j.adaj.2024.05.011__;!!K-Hz7m0Vt54!lbLw9oBzBxIT_8rfcfT_YaykAWLEQLO4OP5ji1G3gDpwPRf55JySUgCU6DWaK7IyUKyj3kj7cPXg$)

Hefferon, R., Goin, D. E., Sarnat, J. A., & Nigra, A. E. (2024). Regional and racial/ethnic inequalities in public drinking water fluoride concentrations across the US. Journal of exposure science & environmental epidemiology, 34(1), 68–76. [https://doi.org/10.1038/s41370-023-00570-w](https://urldefense.com/v3/__https:/doi.org/10.1038/s41370-023-00570-w__;!!K-Hz7m0Vt54!lbLw9oBzBxIT_8rfcfT_YaykAWLEQLO4OP5ji1G3gDpwPRf55JySUgCU6DWaK7IyUKyj3npEp6Pu$)

**We added these two references and updated the text.**

Lines 81-84: Relying on a news article (Hutchinson D, Castronuova C. States move to limit fluoride in water after RFK Jr.'s scrutiny. Bloomberg Law News. 2025 Feb 14 Accessed February 14, 2025) to explain recent shifts in community fluoridation policy weakens the foundation of this section. While political statements may play a role, the authors should address whether there is peer-reviewed epidemiological or toxicological evidence that local and state governments are citing to justify these policy changes.

**As these policy actions occurred in early 2025, no peer-reviewed epidemiological or toxicological literature documenting them is available in the literature. This underscores the significance of the current study to help guide future scientific efforts. No manuscript change was made.**

Methods

Line 97: Considering the STROBE guidelines (Equator Network), there is no clarity regarding the specific study design. Was this an ecological cross-sectional study?

**Yes. We updated the methods section with this information.**

Line 98: Is there a rationale for the specific use/selection of the 2022 data?

**Yes. Calendar year 2022 was the most recent year for which complete data were available from both the CDC WFRS and the U.S. Census Bureau at the time of the analysis.**

Lines 104-107: Linear regression modeling was used. However, there is no mention of the assessment of linear regression assumptions (i.e., linearity, homoscedasticity, normality of residuals, and absence of multicollinearity). Did the authors assume there was a linear relationship? Were non-linear models considered?

**Thank you for this important methodological question. We acknowledge that a full evaluation of ordinary least squares (OLS) assumptions was not conducted prior to the original submission. The use of a proportion outcome bounded on [0, 1] does raise well-recognized concerns about OLS, including the possibility of out-of-range predictions and non-constant variance near the boundaries of the outcome distribution.**

**In preparing this revision, we re-examined the analytic approach and concluded that fractional response regression is the more appropriate method for this outcome [1, 2]. Therefore, all analyses have been reran using fractional logit regression using population weights and robust standard errors. This approach limits predicted values to the [0, 1] interval, does not require distributional assumptions beyond the conditional mean, and accommodates values of 0% and 100% CWF coverage. We did check the conditional mean specifications of all models and found it to be adequate.** **This new analytic approaches led to two main changes in the results (both of which have been included in the revised paper):**

- **% non-white became significant in both the unadjusted and adjusted models;**
- **%<HS education became significant in the adjusted model.**

**We also note that during data preparation for this revision, errors in the vulnerability marker variables were identified and corrected. The revised results presented in this manuscript therefore reflect both the updated modeling approach and the corrected data. Tables and text have been revised accordingly throughout.**

**References:**

**1. Papke LE, Wooldridge JM. J Appl Econom. 1996;11(6):619-632;**

**2. Ramalho EA et al. J Econ Surv. 2011;25(1):19-68.**

Lines 114-117: Descriptives for the variables are presented as aggregated without incorporating statistics at the state level. Could this be added, for example, as a supplementary material?

**This is a great suggestion. We have added choropleth maps for each of the vulnerability markers (Fig. 2-5) to address this.**

Line 114: The software package used to generate Figure I should be specified in the Figure legend.

**Thank you for noting this, the software used is in the figure titles as well as noted in the methods.**

Discussion

Lines 128-129: As a summary of the main findings, the authors concluded that states with higher percentages of Hispanic or Latino individuals had significantly lower percentages of their population on CWF. However, the absence of state-level statistics limits the verifiability of these findings.

**Thank you for this great suggestion. As noted earlier we are including maps with this information in the body of the manuscript.**

Lines 162-164: Similarly to the previous comment, the authors emphasized that efforts are necessary to fully interpret why states with higher proportions of Latino and Hispanic populations had lower CWF prevalence, but the results do not provide descriptives at the state level.

**See above.**

Reviewer #2: General Comments

Could be worthwhile article
But several aspects need additional development/clarification

Specific Comments

Specifics
A. Abstract
1. 40 + 44 + many later places – it is mean “% with” CWF (not level of F=ppm)

**This has been revised.**

2. Lines 40-42 – adjusted?

**Non-adjusted (there was nothing to adjust for in these models). This sentence has been revised to provide clarity.**

3. 50-51 – please replace “may not have” with clearer/stronger statement

**Done.**

B. Intro.
1. 62 – reword “may be influenced” – sounds causal – maybe say “associated with”?

**Done.**

2. 71-75 – shouldn’t this somehow acknowledge that CWF is targeted for larger PWS and not “feasible” in many “very rural” areas

**Thank you. This is an excellent point. We revised the text and provided additional context from reference 13.**

3. 78 – reword “removing… F” – what do you mean?

**This has been revised to “reducing”.**

4. 80 – borne?

**Correct. Thank you for catching this typo.**

5. 85 – missing “the” before first

**This has been fixed.**

6. 85-6 – why listing 3 of 15 in 2026?—expand, reword?

**We have updated this sentence using data from the National Conference of State Legislatures, which documented that at least 21 states introduced bills related to fluoride prohibition during the 2025 legislative sessions.**

7. 91 – why positive?; explain

**We reference previous studies supporting positive associations between % racial and ethnic minorities and % CWF as support for the proposed hypotheses.**

C. Methods
1. 101 – below “the” poverty

**Thank you. This has been fixed.**

2. 105 – among (not between)

**Done.**

3. Is each variable in a separate model – 4?- Or all combined?

**Yes, each is in a separate model. This is clarified in the revised paper.**

4. Any interactions assessed?

**No. We did not hypothesize any interactions, though this would be an interesting topic for future investigation.**

D. Results
1. 116-7 – is age 25+ the variable throughout?; if so, this should be in methods

**Done**

2. 120-1 – not clear the units for the difference

**The unit for the difference is a percent. This has been updated in the paper.**

3. And are the outcomes from regression per % of the explanatory variable or what?

**Please note that the regression model change has led to changes in terminology. Instead of mean differences, we now have average marginal effects (AMEs). However, AMEs are interpreted the same way. We have attempted to clarify their meaning by including a more specific interpretation in the results text and have added a footnote to Table 1.**

**The outcomes from the regression (the AME reported in Table 1) are the change in the percentage of the outcome attributed to the exploratory variable. The classical interpretation is “for every 1% increase in the independent variable (e.g. % non-White at the state-level), on average the dependent variable (% CWF at the state-level) will differ by the AME value presented”.**

**We also note that during data preparation for this revision, errors in the vulnerability marker variables were identified and corrected. The revised results presented in this manuscript therefore reflect both the updated modeling approach and the corrected data. Tables and text have been revised accordingly throughout.**

4. Not clear if 1 variable at a time or all 4 at the same time—if not combined, why not?

**We modeled each variable 1 at a time. Our goal was to isolate the effects of each vulnerability marker rather than assembling a multivariable model. The exception was for the poverty-adjusted models, with poverty modeled as a potential confounder of the association between each vulnerability marker and % CWF.**

5. Table 1
a) Not clear what the units are of outcome and exploratory variables

**The units of the outcome and exploratory variables are all percentages at the state-level.**

b) Bottom right 0.05 - since slightly greater than 0.05 – should either list as 0.06 or show 3rd decimal place (e.g., 0.052 or something)

**Done.**

6. Figure 1 – Needs footnote for source

**We have added the software used to create the map(s) to each figure title as well as noted it in the methods.**

E. Discussion
1. 135-7 – Need to reword/rethink this – explain more – since you did not

**Additional text is provided to expand on this finding.**

2. 140 + 157 – reword “minoritized”

**We replaced it with “marginalized”.**

3. 153 – word “a” missing

**Done.**

4. 151-164 – The whole paper more or less treats the 4 explanatory factors as if they can have direct causative impact on CWF % at the state level. But they cannot. There should be a broader discussion of at least 1-2-3 paragraphs about what determines CWF decisions at the state & community levels to give readers context. Without this, I do not think the article warrants publication.
It should, at a minimum, include discussion of:
1) States with mandatory CWF levels - #, where they are
2) Modes by which other communities decide on CWF policy – usually by elected city officials, sometimes by water board, some by private water companies, (e.g. dominant in NJ – occasionally by public referendum
3) Characteristics from # 1+2 that could be relevant to your analysis.

**These are excellent points. These discussion points are included in the revised paper.**

5. Also, should add more about individuals’ use of bottled water, RO filtration that removes F from water & that ~10-15% of US population (~43 million people) are on non-public water systems

**These are great points and have been added.**

Reviewer #3: While I applaud the authors for drawing attention to an important topic, I have concerns about the somewhat superficial analysis – using state-level CWF data is too broad to derive any of the statements the authors made imho. The authors also rightly point to other studies that some populations ‘shun’ tap water and mostly because of mistrust. Hence, having access to CWF does not necessarily mean gaining its benefits. A few more comments are below:

**Thank you for the thoughtful review. We have addressed your suggestions in the revised paper.**

Title: please add “access to” before CWF.

**Done.**

Abstract: please add (actual) p-values to each statement. “p>0.05” could be p=0.051 or p=1.0…

**Done.**

Introduction:
Line 80 – borne not bore?

**Thank you for noting this typo. It has been corrected.**

Line 88 – of the present study or this study; “the study” is too ambiguous

**This has been revised for clarity.**

Lines 90-91 – “Based on the premise that socioeconomic vulnerability is associated with an increased risk for tooth decay…” There are data on this. Hence, please add at least one reference to support this statement.

**Done.**

Discussion:
Lines 131-132 – “previous studies used more granular county-level data” Why not the present one?

**County-level data with both vulnerability and CWF variables were not available at the time of the analysis. This is one of the priorities for when more granular U.S. data are available, which our team will pursue.**
